# Leveraging remote sensing, geophysical methods and AHP model to determine optimal locations for green hydrogen production on Egypt’s Mediterranean coast

**DOI:** 10.1038/s41598-026-41730-w

**Published:** 2026-03-30

**Authors:** Yasmeen Y. El Hateem, Ahmad I. Diab, Hossam M. El-Sayed, Magdy M. S. El Maghraby, Amr S. Fahil

**Affiliations:** 1 Geology Department, Faculty of Science, Alexandria University, Alexandria, Egypt; 2 National Institute of Oceanography and Fisheries, NIOF, Alexandria, Egypt; 3 Geology Department, Faculty of Science, Tanta University, Tanta, Egypt

**Keywords:** Green hydrogen production, Renewable energy, Vertical electrical sounding, AHP, Bedrock, Marsa Matruh, Environmental sciences, Solid Earth sciences

## Abstract

Global efforts to decarbonize energy systems have intensified the search for renewable alternatives due to reduce rapid climate change, green hydrogen is considered one of the best intriguing solutions. This research integrates remote sensing, GIS, analytic hierarchy process (AHP), and vertical electrical survey to identify optimal locations for production of green hydrogen along Egypt’s Mediterranean coast. Remote sensing and GIS provide spatial and environmental data on surface, AHP supports multi-criteria decision-making, and VES validates the results insights. The methodology employs eight critical parameters: distance to sea, slope, geology, land use/land cover, elevation, distance to roads, wind speed, and air temperature. These parameters were evaluated by utilizing analytic hierarchy process with a consistency ratio of 0.079 which confirms correctness of the weightage method. The resulting suitability map categorizes potential sites into four classes: least suitable, marginally suitable, moderately suitable, and most suitable, which represents 3.5% of the area. Analysis revealed that the northern part of Marsa Matruh represents the most favorable location for green hydrogen production. Additionally, a geoelectrical survey using eleven vertical electrical soundings (VESs) with Schlumberger configuration validated the surface findings and provided crucial subsurface information, suggesting dolomitic limestone as the optimal bedrock for facility construction which found at a depth ranging between 1.3 and 47 m with resistivity values ranging from 185.7 to 2251 Ω m. This study offers a thorough framework for the strategic advancement of green hydrogen production in Egypt, supporting the country’s sustainable energy transition goals.

## Introduction

The consistent rise in global energy demand, driven by population growth, elevated living standards, and industrial expansion in developing nations, presents significant environmental challenges^1,2^. Currently, greater than 95% of significant energy needs are satisfied by fossil fuels, leading to huge greenhouse gas emissions that intensify global warming problem and environmental deterioration^3,4^. Global initiative has been established to mitigate these emissions and control the rise in the worldwide average temperature to less than 3 °C^5–7^. Therefore, renewable energy is the best solution for replacing nonrenewable energy sources, and one of these renewable sources is hydrogen.

Hydrogen is well positioned to store and transfer renewable energy, taking a vital role in the world’s energy transformation^8^. The classification of hydrogen varies according to production methods and environmental impacts, with designations including blue (reforming with carbon capture from natural gas), gray (reforming from natural gas), brown (gasification from lignite), black (gasification from bituminous coal), and green (electrolysis from water)^9,10^. As the most environmentally beneficial option, green hydrogen is generated using renewable electricity that comes from renewable energy sources to split water into hydrogen and oxygen that leads to zero carbon emissions while production^11^. Hydrogen possesses many advantages, for example, it is renewable, clean, non-polluting, adaptable, and storable. Hydrogen reserves are plentiful, also their unit calorific value is relatively high in contrast to other fuels^12,13^.

Renewable hydrogen has diverse applications across multiple sectors, including renewable energy production for transportation, industrial processes such as steel manufacturing and petroleum refining, and chemical production like ammonia and methanol^14^. However, despite its numerous benefits, producing and managing hydrogen especially, green hydrogen presents significant challenges. These include the high costs of electrolysis equipment, the technical complexity of integrating intermittent renewable energy sources, and the substantial energy requirements for storage, compression, and transportation of hydrogen. Additionally, hydrogen’s high flammability and low ignition energy necessitate specialized handling processes and safety systems^15^.

Alongside global initiatives for decarbonization and the transmission of renewable energy, Egypt aims to generate green hydrogen utilizing its plentiful clean energy resources, such as wind and solar energy. As technology continually advances to enhance the production process, it has become essential to identify the most suitable location for production. Egypt has favorable circumstances for production. Marsa Matruh is one of the ideal areas because of its geographical location along the northwest coastline of Egypt which provides sea water for electrolysis process and exportation due to possessing and Gargoub port that links Egypt with Europe. Additionally, the port town’s location on the mainland route between Alexandria and Cyrenaica, as well as its connection to the primary route southwest to the oasis of Siwa made it a key junction for transportation and trade, both inland and at sea^16^. It also has suitable climatic conditions to produce solar and wind energy needed to produce the electricity used to operate electrolyzer devices as well as possessing vast areas for establishing this project.

Although spatial analysis for renewable energy planning has advanced, no previous study in Egypt has combined remote sensing, geographic information systems, and the analytic hierarchy process (AHP) with field-validated geophysical data such as vertical electrical sounding for green hydrogen site suitability assessment. While prior studies^17,18^ have demonstrated the value of integration of satellite image analysis with GIS mapping enhances the strategic approach and improves understanding of results in addition to statistical methodologies like the analytic hierarchy process that increase the accuracy of results. However, this combination method is missing in the Egyptian context for determining the best location for green hydrogen production.

**Fig. 1:**
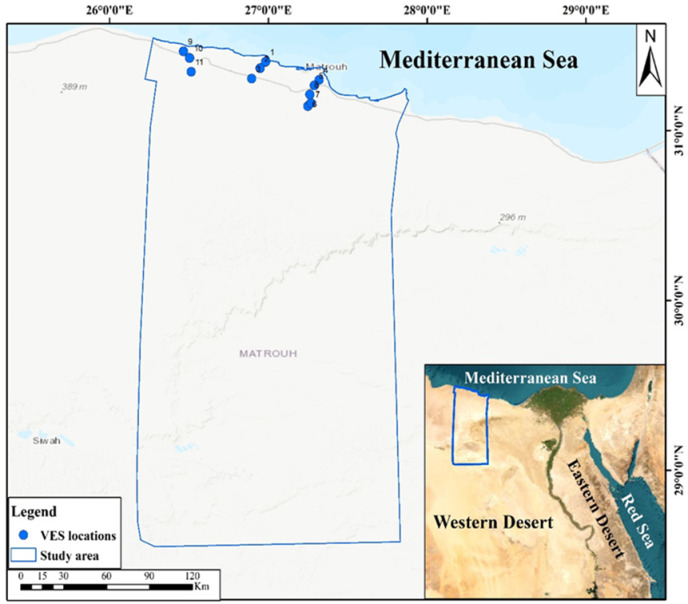
Location of the vertical electrical soundings distribution in the study area (*The map generated by* Esri ArcGIS 10.5; https://www.esri.com).

Therefore, this study addresses this gap by identifying optimal locations green hydrogen production on the northwestern coast of Egypt especially, Marsa Matruh city (Fig. 1) through an integrated remote sensing satellite imaginary, GIS, AHP methodology and field validation using vertical electrical sounding (VES) surveys.

## Study area and tectonic setting

Matruh Governorate represents one of the most promising locations on Egypt’s northern coast, particularly the Marsa Matruh city, which is pivotal for upcoming sustainable development projects. The population of Marsa Matruh reached 241.625 by the year 2024. It is situated 240 km west of Alexandria and 222 km east of Sallum, along the main highway connecting the Nile Delta and the Libyan border together and another highway extends in the south towards Siwa Oasis and Bahariya Oasis. Marsa Matruh International Airport and Gargoub port have granted the city significant logistical value, positioning it as an attractive place for industry, investment, and international trade. The research region ranges from latitude 28° 33′ N to 31° 32′ N and longitude 26° 10′ E to 27° 52′ E (Source, ArcGIS) within the Matruh Basin.

**Fig. 2:**
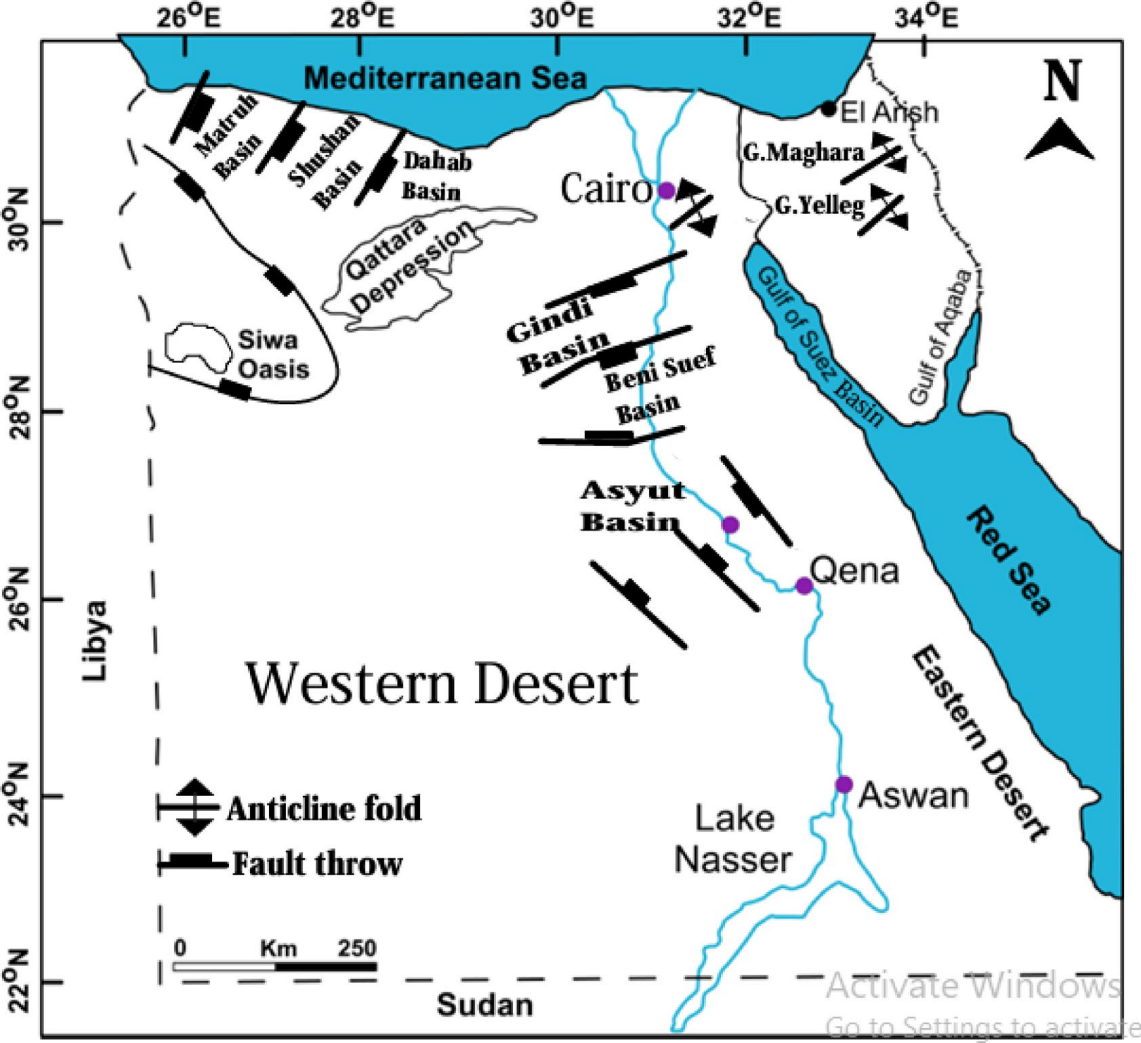
Global view of tectonic setting of Egypt showing major sedimentary basins, including Matruh Basin, and structural features (modified after Egyptian General Petroleum Corporation^24^).

Tectonically, Matruh Basin (Fig. 2) constitutes a segment of a sequence of intracratonic rift basins located along the northern African passive margin^19^ that formed during the Permian to Early Jurassic periods in the northern Western Desert of Egypt^20–22^. These basins typically follow either NE SW or ENE-WSW orientations^23^, with the Matruh basin distinguished as a predominantly NNE-oriented formation^19^. The Matruh Basin’s structural framework has a direct influence on regional geotechnical engineering decisions. Planning for construction projects necessitates an accurate assessment of rock mass behavior under stress, slope stability, and probable tectonic activity threats. This makes structural studies a crucial component of determining the engineering viability of any location inside the basin.

## Materials and methods

### Remote sensing

This research employs a comprehensive methodology to determine the best places to produce green hydrogen by evaluating eight key parameters including land use/land cover (LULC), climate data (Air temperature, wind speed), elevation, slope, geology, distance to roads, and distance to sea. The parameters were selected for the weighted overlay analysis and evaluated using ArcGIS Desktop 10.8 software. All data processing, including coordinate system projection and raster resampling was performed. The methodology involved the following structured approach:

#### Data collection

To create the thematic layers required for analysis, high-resolution multispectral imagery from different sources were utilized. The specific layers were derived as follows:

- Land use/land cover map: Generated from Sentinel-2 imagery at 10 m resolution. LULC map was created by unsupervised classification producing 4 clusters (water, bare ground, built-up area, and rangeland) to identify land availability for establishing green hydrogen production facilities such as infrastructure for producing hydrogen and establishing solar and wind energy projects.
- Geology: Geological data was acquired from the United States Geological Survey (USGS). It was used to identify rock units that could influence site selection and determine the age and characteristics of the bedrock.
- Elevation: Digital elevation models (DEMs) were derived from Copernicus GLO-30 data accessed through Open Topography at resolution of 30 m. Elevation data are vital for understanding the topography of the site, which can affect the feasibility of infrastructure development.
- Proximity maps: Transportation infrastructure data (railways and roads) was obtained to evaluate connectivity for hydrogen transportation and distribution. Proximity to the sea was derived from the Marsa Matruh coastline shapefile to determine distance from sea for getting water for electrolysis.
- Climate data: Information on air temperature and wind speed was collected from WorldClim website and NASA power, respectively. These data are crucial for assessing the potential for wind and solar energy sources, that considering the main sources to generate electricity that is used in the electrolysis process at 2.5-min spatial resolution (~ 21 km^2^ at the equator) for air temperature map and at 10 m (m/s) for wind speed map.
- Slope: was extracted from DEM. It’s vital for determining the feasibility of constructing green hydrogen infrastructure in a specific area to determine the gradient of the region.

#### Data processing

The raw satellite imaginary was processed by utilizing (ArcGIS) software through the following steps:

- Layer generation: Each thematic layer was produced through specific algorithms and models. Air temperature and wind speed were calculated using inverse distance weighted (IDW) interpolation as it’s an effective method for determining spatial variability of climate data, making it appropriate for our investigation since it doesn’t require sophisticated assumptions regarding data distribution. The Euclidean distance function was applied for proximity analysis, and unsupervised classification techniques were employed to generate the LULC map.
- Data integration: The layers generated were integrated into a comprehensive GIS database, with standardization applied to ensure compatibility and consistency across the dataset.

#### Site suitability analysis

Leveraging a multi-criteria decision-making (MCDM) to determine the optimal sites for green hydrogen production as illustrated through the following procedures.

##### Criteria weighting using AHP

Each of the eight parameters was given a weight depending on their relative relevance for green hydrogen production. Weights of each parameter were determined by using pairwise comparisons method that resulted from analytic hierarchy process (AHP) as illustrated in Table 1. This quantitative scale, which followed the technique created by Saaty^25^, varied from 1 to 9 based on the relative relevance of each parameter as shown in Table 2. In spatial decision-making, AHP represents a robust method for allocating weights to multiple parameters^26,27^. The AHP also uses a mechanism to examine the consistency of the experts’ scoring and eliminate any personal bias^28^. The scale in Table 2 that defined by Saaty assists four experts in scoring the relative importance of each parameter compared to each other. The parameters’ final weights were computed by normalizing each eigenvector of the reciprocal ratio matrix. Before finalizing the parameter weights, the consistency of experts’ scoring was tested utilizing these equations^29^ that developed by^25,30^:

$$Consistency\;index\;\left( {{\mathrm{CI}}} \right) = \frac{\lambda max - n}{{{\mathrm{n}} - 1}}$$

$${\mathrm{Consistency}}\;{\mathrm{ratio}}\;\left( {{\mathrm{CR}}} \right) = \frac{{{\mathrm{CI}}}}{{{\mathrm{RI}}}}$$

where λ max stands for the maximum approximation of eigenvalue, the number of criteria is indicated by n, and the random consistency index for n criteria is called RI. The CR value ought to be below 0.1 for acceptable consistency. The final step involved applying weights derived from expert opinions to reclassified analyses in the ArcGIS environment^31^.

**Table 1:** Pairwise comparison matrix.

	Distance to sea	Air temperature	Wind speed	LULC	Slope	Elevation	Geology	Distance to roads	Weight %
Distance to sea	**1**	3	3	3	4	4	4	4	30
Air temperature	1/3	**1**	2	3	3	3	3	3	18.7
Wind speed	1/3	1/2	**1**	3	4	4	4	4	18.2
LULC	1/3	1/3	1/3	**1**	2	2	3	3	10
Slope	1/4	1/3	1/4	1/2	**1**	3	3	3	8.6
Elevation	1/4	1/3	1/4	1/2	1/3	**1**	2	3	6
Geology	1/4	1/3	1/4	1/3	1/3	1/2	**1**	3	5
Distance to roads	1/4	1/3	1/4	1/3	1/3	1/3	1/3	**1**	3.5

**Table 2:** AHP pairwise comparison scale^25^.

Intensity	Definition	Explanation
1	Equal importance	Two activities contribute equally to the objective
3	Moderate importance	Judgment slightly favors one activity over another
5	Strong importance	Judgment strongly favors one activity over another
7	Very strong importance	An activity is favored very strongly over another
9	Extreme importance	Favoring one activity over another is of the highest affirmation
2, 4, 6, 8	Intermediate values	A compromise is needed

##### Layer overlay analysis

**Fig. 3:**
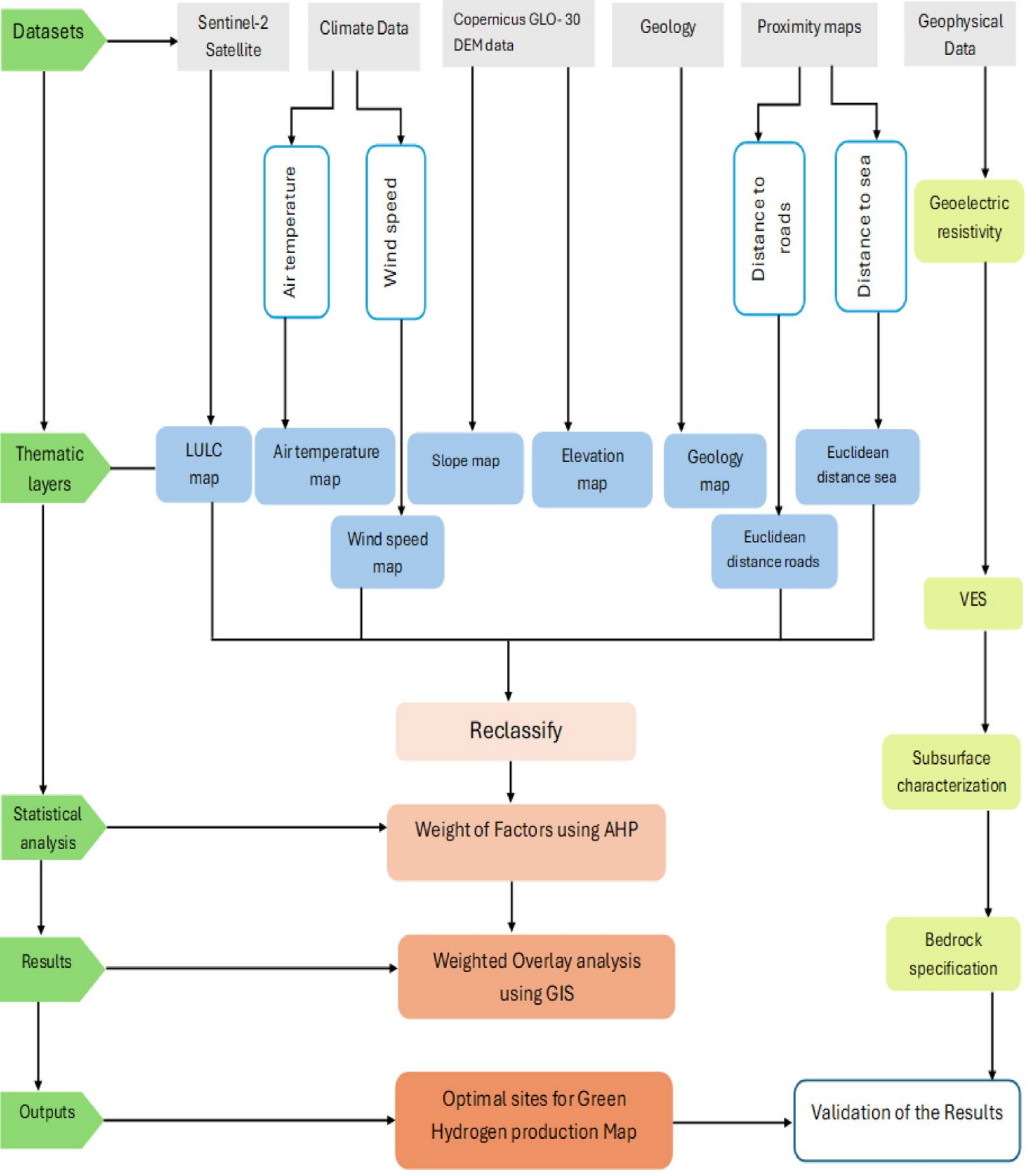
Methodological flow chart.

Standardization of criteria: Standardization involved converting vector layers to thematic layers using ArcGIS software’s reclassify tool then the raster lost dimension and measurement units^32^. The weighted layers were overlaid in the GIS environment to create a composite suitability map. All parameters were reclassified into raster format with four classes (Fig. 3) on scale ranges 1–4 that 1 has the least value and 4 represent the highest value, but geology map was reclassified into three classes according to local geological history in this study area that shows three main rock units only represented by (Miocene, Holocene, and Quaternary). The overlay analysis combined the layers using a weighted sum approach, with each pixel’s suitability score calculated as the sum of the weighted values from individual layers. The formula used for calculating the final suitability index is:

$${\mathrm{S}} = \left( {{\mathrm{W}}_{1} \times {\mathrm{X}}_{1} } \right) + \left( {{\mathrm{W}}_{2} \times {\mathrm{X}}_{2} } \right) + \left( {{\mathrm{W}}_{3} \times {\mathrm{X}}_{3} } \right) + \cdots + \left( {{\mathrm{W}}_{{\mathrm{n}}} \times {\mathrm{X}}_{{\mathrm{n}}} } \right)$$

where S = Final suitability index, W_n_ = Weight assigned to factor n (based on its importance), and X_n_ = Normalized score of factor n (based on reclassified raster values).

##### Site selection

The composite suitability map was classified into different categories (most suitable, marginally suitable, moderately suitable, and least suitable). Sites with the highest suitability scores were identified as optimal locations for green hydrogen production.

### Vertical electrical sounding (VES) survey

Vertical electrical sounding (VES) is specifically designed to study vertical variations in underlying rock resistivity^33^. This technique operates on the principle of injecting current from the surface into the subsurface using a transmitter through stainless steel electrode and recording the potential difference response using a receiver which is done by a copper electrode or at a well-conductive point within the soil or rock^34^. The current is injected into rock/soil formations and flows through pores or minerals, with ease of flow indicating conductive characteristics and resistance to flow suggesting resistive properties^35^. The resistivity method has diverse applications in mineral exploration, environmental investigation, groundwater identification, and geotechnical assessment^36^.

This study used Syscal Pro electrical resistivity meter that used a technique of Schlumberger array configuration that included eleven VES (Fig. 1), in which all four electrodes are aligned linearly and with half-current electrode spacing (AB/2) that ranged from 1.5 to 400 m. The current electrodes were repositioned while the potential electrodes remained stationary, being adjusted only when signal strength diminished during each measurement after these processes, apparent resistivity data were acquired and evaluated to determine each geoelectric layer’s resistivity and thickness^37^. All data were processed by using IX1D inversion software to obtain subsurface layers model including resistivity values, thicknesses, and depths of the various strata. The 1-D inversion approach is an iterative procedure designed to determine a layered earth model (layer thickness and resistivity) that reduces the discrepancy between the field-measured apparent resistivity values and the theoretical values derived from the model. Starting with an initial model, the software automatically updates the model parameters until the best fit is found, as indicated by the root mean square error (RMS). The inversion results were validated using multiple approaches like verifying data consistency by comparing inverted layer depths to surrounding geological information as illustrated in the result section in addition to statistical and geological checks by accepting the final model with minimal RMS error (< 10%) and a good visual fit on the curve match plot.

## Results and discussion

### Remote sensing results

#### Proximity to sea

**Fig. 4:**
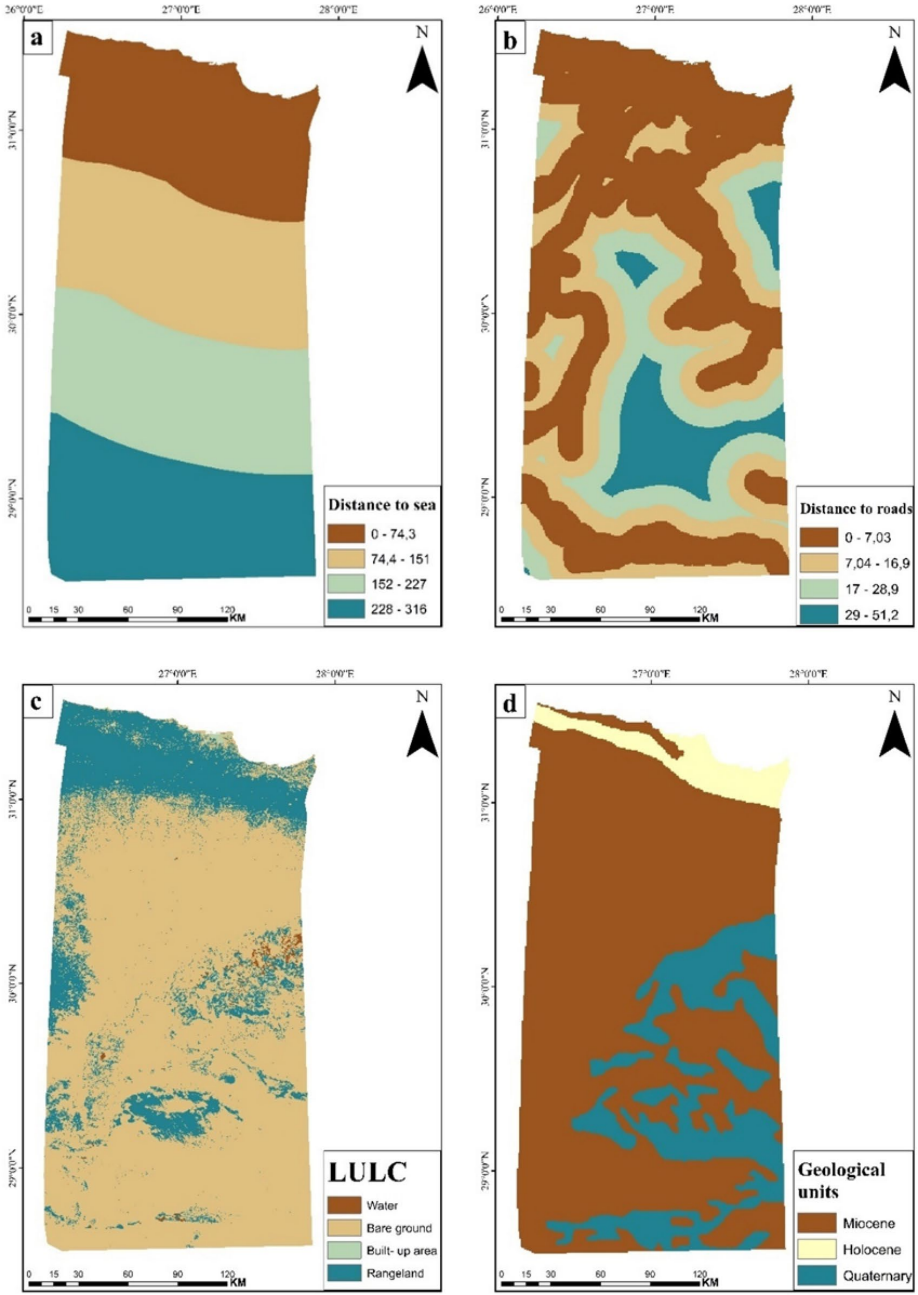
Thematic layers of (**a**) Euclidean distance to sea shows the distance from each location to the coastline, (**b**) Euclidean distance to roads indicates proximity to transportation networks, (**c**) LULC classifies the region into different categories (water, bare ground, built up area, and rangeland) based on unsupervised classification, and (**d**) Geology represents three different geological formations in the area. (*The map generated by* Esri ArcGIS 10.5; https://www.esri.com).

Marsa Matruh’s location on Egypt’s northwestern Mediterranean coast provides excellent access to water (Fig. 4a). Water is an essential component in the electrolysis process that produces green hydrogen. Therefore, the proximity of optimal sites to available waterbodies is of highest importance^29^. Additionally, the area represents a strategic location for various industries and international trade due to the new Gargoub Port, which connects Egypt with European ports and would facilitate the export of green hydrogen to European markets.

#### Proximity to roads

The Euclidean distance function was applied to generate proximity to roads map by merging road and railway layers into a single transportation network file (Fig. 4b). The proximity to roadways represents the relevance of hydrogen transportation and its accessibility. Areas near roadways often have greater transportation infrastructure, making them more accessible for numerous activities^38^.

#### Land use/land cover (LULC)

Decision-makers in a variety of industry sectors and emerging countries are finding land use/land cover mapping to be an increasingly helpful tool. LULC is considered one of the key parameters for the installation of green hydrogen infrastructure and solar power plants and wind farms.

The analysis identified four primary land use classes in the region including rangeland, water bodies, built-up areas, and bare ground (Fig. 4c), based on high resolution Sentinel-2 satellite imagery. The rangeland and bare ground are most suitable for establishing the infrastructure.

#### Geology

Geological history is a vital element for exploring any region as it shows the rock units in the region, in addition to the nature of these rocks and the geological processes affecting them. In this study area, three geological units are found including, Tertiary deposits cover the largest part of the area, particularly Miocene formations comprising a basal clastic section overlain by a carbonate unit (Fig. 4d). Holocene deposits include sabkha formations, while Quaternary deposits (wadi and playa deposits) are predominantly distributed across the northern sites of the area.

#### Digital elevation model (DEM)

**Fig. 5:**
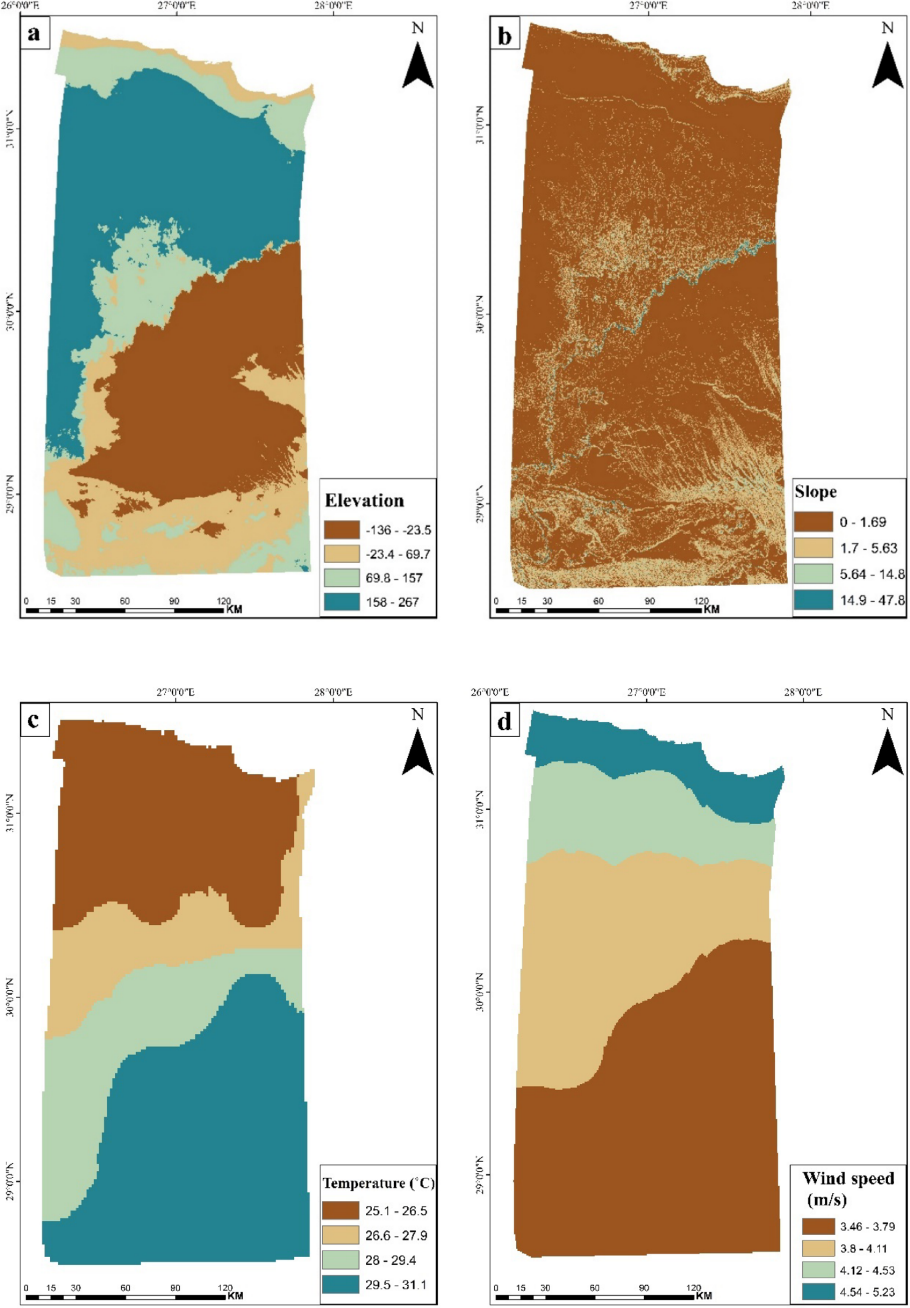
Thematic layers of (**a**) Elevation (m) displays terrain height which affects construction and environmental conditions, (**b**) Slope values in degrees showing the spatial distribution of slope angles, (**c**) Air temperature illustrates the spatial variation of ambient temperature which influences energy demand, and (**d**) Wind speed helps in assessing wind energy potential. (*The map generated by* Esri ArcGIS 10.5; https://www.esri.com).

Copernicus GLO-30 data was used to extract the digital elevation model at a 30-m resolution (Fig. 5a). DEMs have many applications in different fields such as hydrologic and geological assessments, hazard monitoring, natural resource exploitation, and agricultural management^39^. For this research, the DEM played a crucial role in site selection for setting up green hydrogen manufacturing plants and related renewable energy infrastructure, such as wind farms and solar panels. The analysis revealed that elevations within the area ranged from − 135 to 267 m above sea level, with the lowest values corresponding to the Qattara Depression which is considered Egypt’s deepest (− 145 m) and largest (~ 45,000 km^2^) depression that is located in the northern portion of the Western Desert^40^. The further away from the low-lying areas, the better for construction that the project isn’t affected by the risk of torrents and floods in addition to higher elevation sites were thought to be more appropriate for the selection of optimal areas for solar-based green hydrogen production because of the benefits they provide for PV solar panels^29^.

#### Slope

Slope analysis is a critical parameter in site selection for establishing power projects. Slope maps were used to know the gradient of the area. Solar arrays are most suited for places with gentle slopes due to their flexibility in design and placement, as well as their ability to receive optimum sunshine throughout the day. Additionally, slope evaluation is important for developing infrastructure for green hydrogen production, including storage and delivery systems^41^. The northern parts of study area possess a gentle slope (Fig. 5b) which is suitable for establishing green hydrogen infrastructure.

#### Air temperature and solar radiation

Solar power represents a critical factor in green hydrogen production site selection, as it provides the electrical energy required for operating electrolyzer devices that electrolysis water to separate H_2_O into O_2_ and H_2_. The Inverse Distance Weighted (IDW) interpolation results for air temperature demonstrated that the entire study area receives substantial solar radiation (Fig. 5c), making it suitable for photovoltaic system installation. This favorable condition could incentivize the Egyptian government to develop solar energy infrastructure along Egypt’s northwest coast, potentially creating employment opportunities, generating green electricity, and promoting sustainable development^42^.

In addition to air temperature, solar radiation is also a crucial factor for establishing PV power plants. The construction of solar powered plants needs to access solar radiation in the region. Egypt is marked by its geographical location near the equator, which puts it constantly exposed to a high amount of solar radiation during the whole year. In Marsa Matruh, solar radiation levels vary from 122.47 Wh/m^2^/day in December to 322.86 Wh/m^2^/day in June. Solar radiation is lowest between November and February, and strongest from April to October (Atallah et al. 2025) which indicates the importance of integration between solar and wind power projects.

The amount and intensity of solar radiation in a specific area affects the efficiency and viability of solar power generation, affecting green hydrogen production. Analyzing solar radiation levels in a potential area can help determine the quantity of renewable energy available for green hydrogen production^41^.

#### Wind speed

Marsa Matruh’s location on the northern coast of Egypt makes it receives high wind speeds through the whole year. The inverse distance weighted (IDW) interpolation analysis indicated maximum wind speeds of 5.22 m/s in the northern section of the study area (Fig. 5d), representing favorable conditions for wind energy development. Recent advancements in wind turbine technology have enabled the establishment of larger wind farms in coastal and oceanic regions for increased energy generation^43^. The integration of wind and solar energy can significantly enhance electric power generation for electrolyzer operation as Egypt receives a large amount of solar radiation during the year due to its closeness to the equator and Marsa Matruh also has high rates of wind speeds as coastal city.

### Delineation of green hydrogen production site selection

**Fig. 6:**
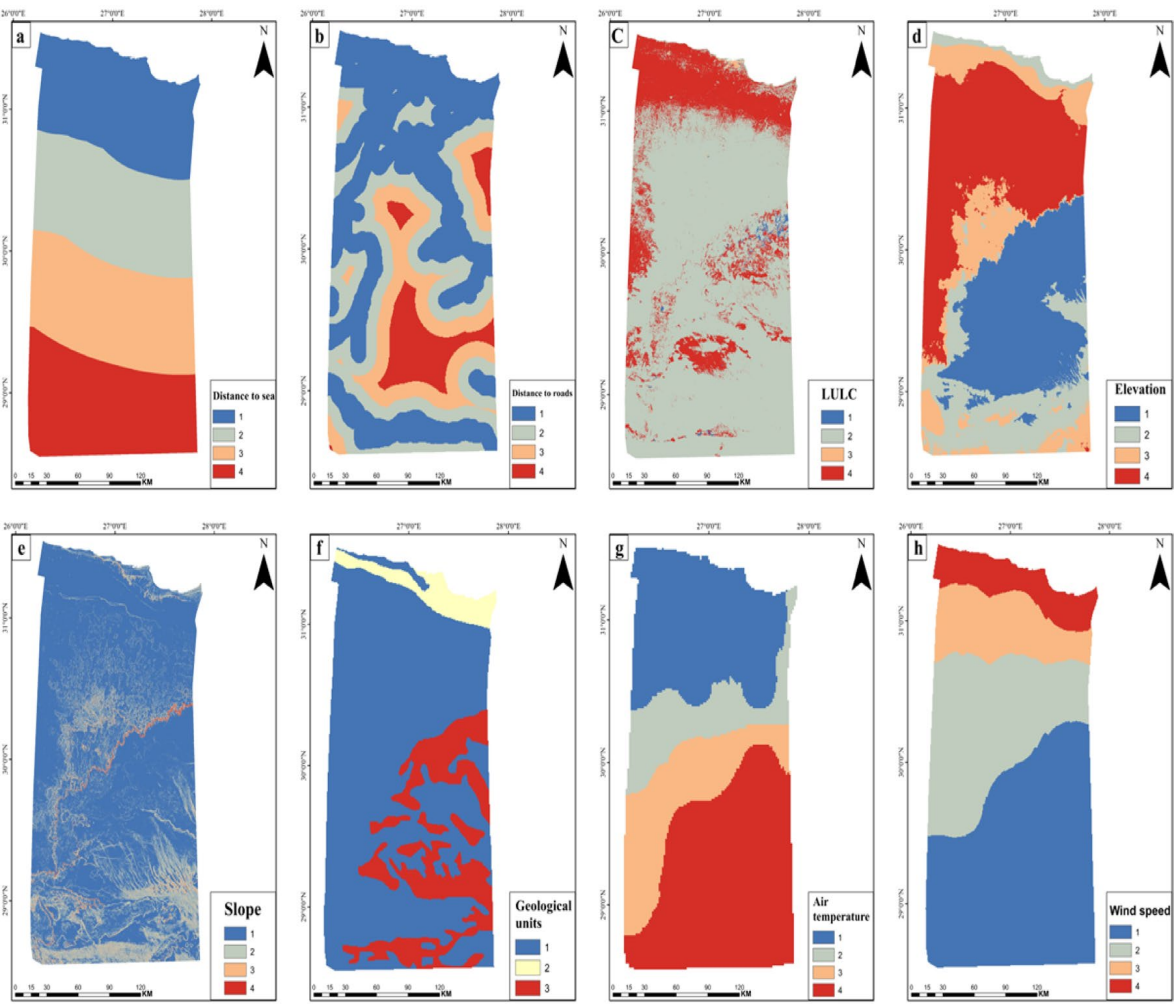
Reclassified thematic layers were integrated using AHP to assess site suitability for green hydrogen production: (**a**) Euclidean distance to sea, (**b**) Euclidean distance to roads, (**c**) LULC, (**d**) Elevation, (**e**) Slope, (**f**) Geology, (**g**) air temperature and, (**h**) Wind speed. (*The map generated by* Esri ArcGIS 10.5; https://www.esri.com).

**Fig. 7:**
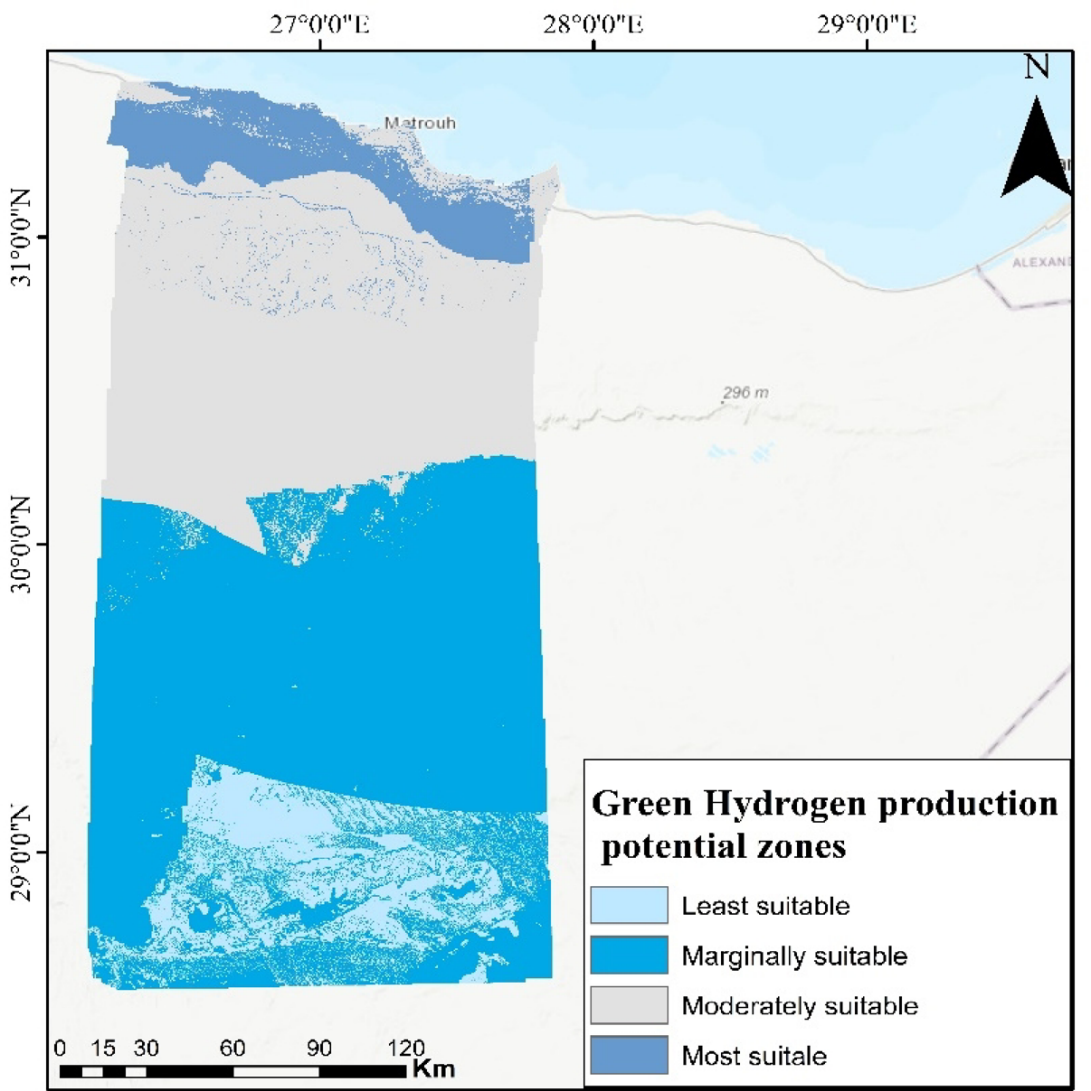
Green Hydrogen production suitability index map obtained from 8 integrated remote sensing parameters and identified 4 potential zones ranging from least suitable in the south to most suitable in the north. (*The map generated by* Esri ArcGIS 10.5; https://www.esri.com).

The pairwise comparison approach was employed to categorize and estimate the weight of parameter^44^. Table 3 displays the allocated scores and weights for each parameter resulting from analytic hierarchy process methodology, with a consistency ratio (CR) of 0.079, indicating high reliability in the weighting procedure. The eight parameters are reclassified into four classes except geology into 3 classes because it consists of 3 criteria (Miocene, Holocene, Quaternary), therefore after reclassifying it has 3 classes as shown in Fig. 6 and then the reclassified maps were used to perform weighted overlay analysis to create a suitability map for green hydrogen production. The comprehensive analysis of parameters identified locations with high suitability for green hydrogen production within the study region, categorized into four distinct zones: least suitable, marginally suitable, moderately suitable, and most suitable represents about 47.4% of the region, respectively (Fig. 7).

**Table 3:** Analytic heirarchy’s parameters weight.

Parameter	Class	Score	Weight (%)
Distance to sea	0–74.3	4	30
	74.4–151	3	
	152–227	2	
	228–316	1	
LULC	Water	1	10
	Bare ground	3	
	Built-up area	2	
	Rangeland	4	
Geology	Miocene	3	5
	Holocene	1	
	Quaternary	4	
Air temperature	25.1–26.5	4	18.7
	26.6–27.9	3	
	28–29.4	2	
	29.5–31.1	1	
Wind speed (m/s)	3.46–3.79	1	18.2
	3.8–4.11	2	
	4.12–4.53	3	
	4.54–5.23	4	
Slope	0–1.69	4	8.6
	1.7–5.63	3	
	5.64–14.8	2	
	14.9–47.8	1	
Distance to roads	0–7.03	4	3.5
	7.04–16.9	3	
	17–28.9	2	
	29–51.2	1	
Elevation (m)	− 136 to − 23.5	1	6
	− 23.4 to 69.7	2	
	69.8–157	4	
	158 to 267	3	

### Vertical electrical sounding (VES) survey results

The resistivity measurements were processed to develop a subsurface model for identifying bedrock layers, with readings used to characterize subsurface features and estimate rock composition. The results of one-dimensional (1D) inversion data processing were interpreted depending on data from nearby wells to create a comprehensive model for the subsurface layer of the region. All VESs were interpreted and only 3VESs were mentioned here as examples for other VESs and the interpretation of the subsurface lithology at each sounding point is shown in Table 4.

**Table 4:** Resulted parameters of the geoelectrical survey.

Layers	Lithology	Thickness (m)	Resistivity (Ω m)
1	Wadi deposits	0.4–4.5	12.9–470.3
2	Dolomitic limestone	0.8–14.4	185.7–2251
3	Marly limestone	2.2–13.8	28.2–138.2
4	Clay saturated by saltwater	1.1–47	0.1–7.9
5	Fossiliferous limestone	1.7–66.7	49.5–268.3
6	Saturated fractured limestone	14.3–57.2	10.3–62.2
7	Limestone intercalated with clay saturated by saltwater	–	8.5–13

**Fig. 8:**
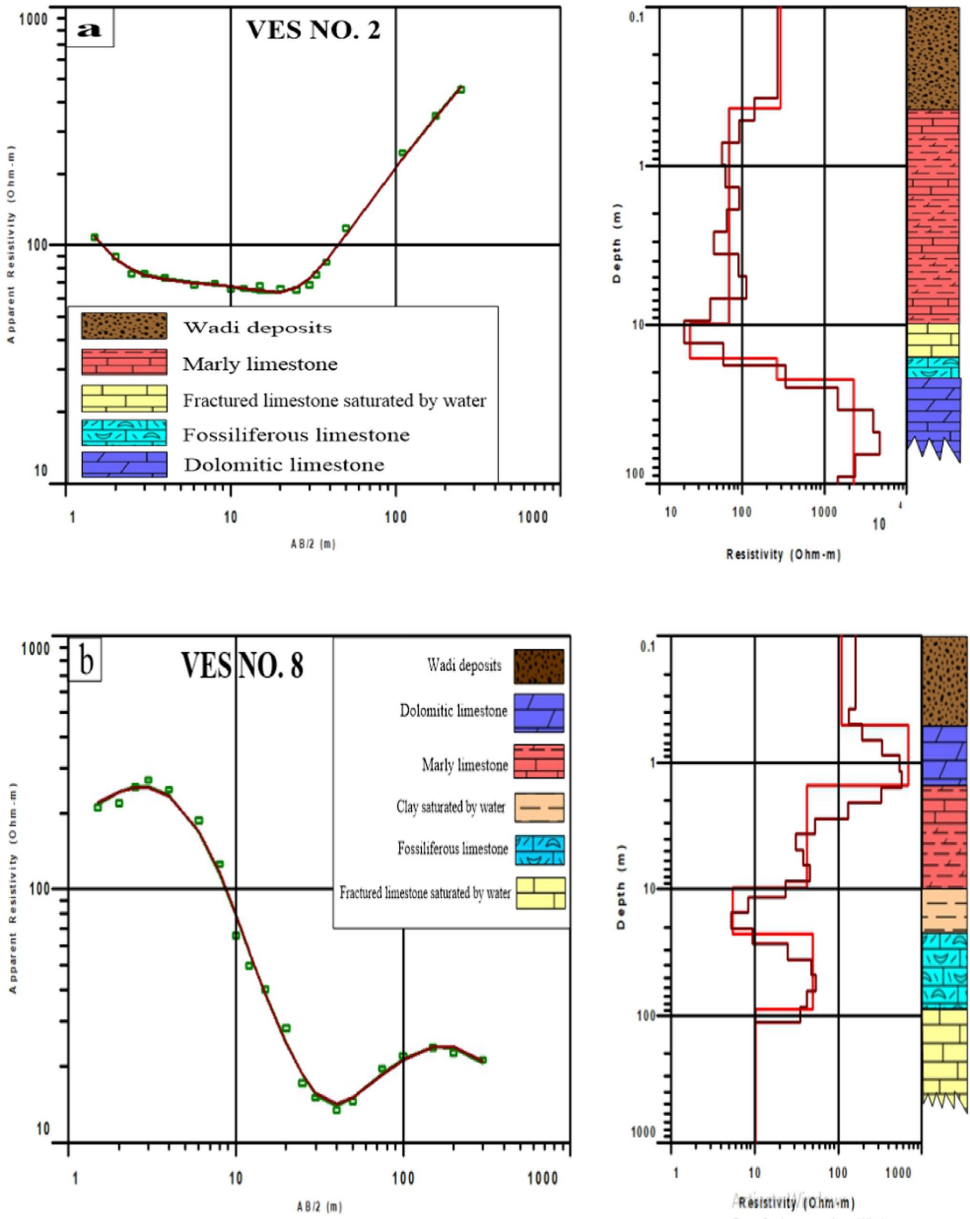

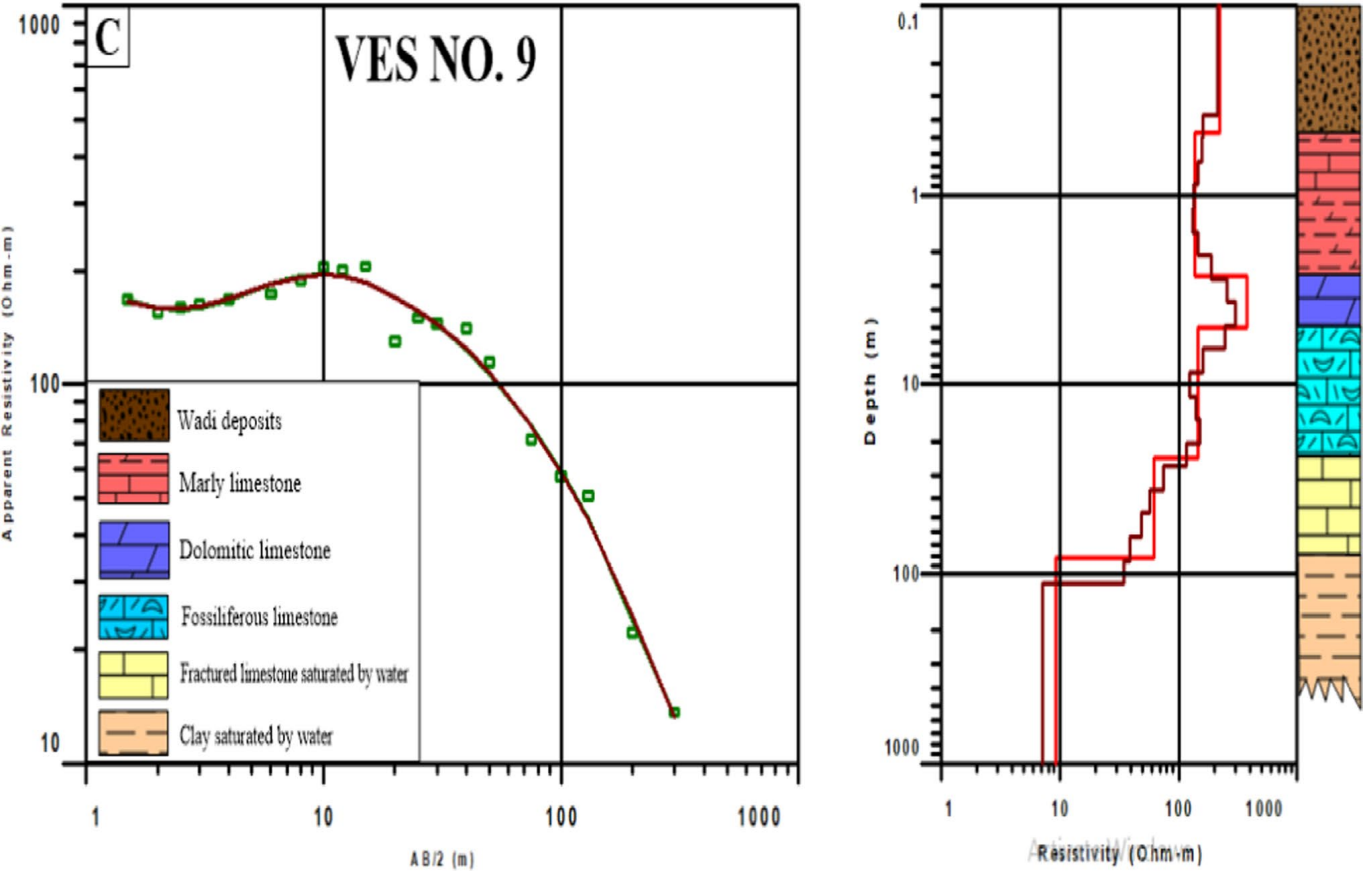
Interpreted models of (**a**) VES No. 2, (**b**) VES No. 8, and (**c**) VES No. 9. (The models figure genarated from IX1D v3 software; http://www.interpex.com).

In VES. 2, calibrating it with field data from surrounding drilled wells according to Barseem et al.^45^, there were five different units in this model: surface wadi deposits, marly limestone, and saturated fractured limestone, fossiliferous limestone, and dolomitic limestone (Fig. 8a). In VES. 8, calibrating it with surrounding available data according to Gemail et al.^46^ there were six different units: Wadi deposits, dolomitic limestone, marly limestone, clay saturated by saltwater, fossiliferous limestone and saturated fractured limestone (Fig. 8b). In VES. 9, calibrating it with drilled well according to El-Sayed and Elgendy^44^ there were six different units: wadi deposits, marly limestone, dolomitic limestone, fossiliferous limestone, saturated fractured limestone, Limestone intercalated with clay saturated by saltwater (Fig. 8c).

The bedrock of infrastructure should be hard and can endure heavy weights. The higher the resistivity, the greater the hardness. Based on these results, dolomitic limestone possesses the highest resistivity values varying from 185.7 to 2251 Ω m and found at a depth ranging between 1.3 m in VES number 6 and 7 and 47 m in VES number 3 that may be suitable as a bedrock.

The geophysical results indicate a clear spatial variation in the subsurface strata across the study area as in VES number 2 which locates in the middle of the northern part of study area the dolomitic limestone that represents a bedrock layer is found at deep depth (~ 20 m) unlike VES 8 and 9 that are found in the eastern and western part of the region, respectively, dolomitic limestone appears at shallow depths despite belonging to the same depositional environment. As the VESs are sparse in the region and the depth of the bed rock in the middle is large and seems appropriate, it seems to be initial indicators for the most suitable region for establishing green hydrogen infrastructure.

The integration of GIS and VES in this study is critical because it establishes a synergistic framework for optimal green hydrogen site selection. GIS provides information about factors that affect the selection of sites on surface. However, GIS alone can’t verify subsurface conditions. The importance of this integration is VES validates the results obtained from GIS and moves from theoretical suitability to ground-trusted feasibility which reduces exploration risk and capital cost by ensuring selected sites meet all critical geospatial and geophysical criteria for large-scale green hydrogen production.

This study demonstrated that hybrid renewable energy systems provide a dependable supply of green hydrogen to advance a sustainable and decarbonized energy sector. During the winter of December, January, and February, wind turbines generate more power than PV due to high wind speeds. In other months, however, the PV electricity is at its peak. As a result, the annual amount of power generated with a high PV percentage exceeds WT^47^. Egypt produces 8.48 kg/m^2^ of hydrogen for the PV system, while the production of hydrogen for the WT system is 1.31 kg/m^2^^48^. Thus, the integration between PV and WT in this project will enhance the production capacity of green hydrogen sustainably. The site that is economically feasible for power generation is characterized by an annual average wind speed of 20 km/h at a height of 30 m, and a power density of 150 W/m^2^^49^. Marsa Matruh is characterized by these suitable conditions as its annual average specific wind power of 180–230 W/m^2^ at a height of 30 m above ground. At heights of 30 and 50 m, the average wind speed per year is estimated to be 6.98 and 7.93 m/s^50^.

**Fig. 9:**
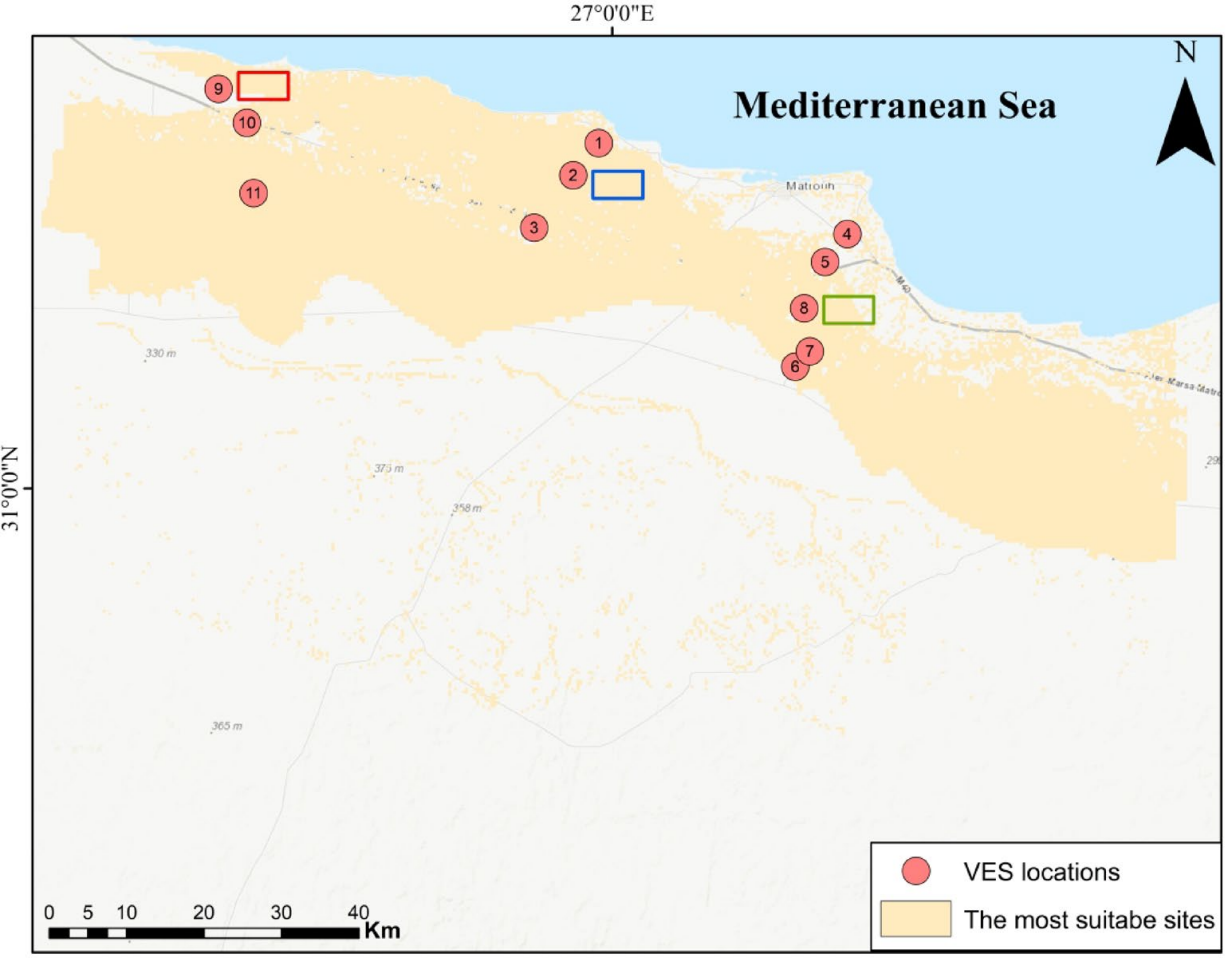
Map of the most suitable sites in the study area. The map illustrates VES locations and the depths of dolomitic limestone at VESs number 2, 8, and 9. The red and green boxes indicates to the presence of dolomitic limestone at shallow depths, but blue box shows the depth of dolomitic limestone at depth of 20 m that can be recommended area for establishing green hydrogen infrastructure. (*The map generated by* Esri ArcGIS 10.5; https://www.esri.com).

Ultimately, it has been found that the most suitable land is near major roads, transmission lines, and urban areas (Fig. 9). Also, it is close to Gargoub port that links Egypt with Europe which will help in exporting of green hydrogen to Europe and other countries that will enhance the economy and national income of Egypt.

## Conclusion

This paper sought to propose a realistic strategy for determining land availability of green hydrogen production for Egypt’s Mediterranean coast by combining AHP technique with RS and GIS tools. The results can support the development of the green hydrogen sector, preserve energy for upcoming requirements, and support regional sustainable initiatives. As the worldwide demand for clean energy solutions grows, strategies that incorporate diverse data sources and analytical tools will become more relevant for accelerating the growth of renewable energy sources and supporting the shift to economies with lower carbon emissions. The International Energy Agency^51^ claims that, Egypt is a prospective hub for green hydrogen production, aiming to contribute 8% to the world hydrogen market and by 2050 the levelized cost of hydrogen (LCOH) will be1.7 $/kg.

The integration of satellite imaginary and validating the results with geophysical data and AHP is a novel way to select the best places for green hydrogen production. The synergy between these complementary techniques, combining both surface and subsurface viewpoints, significantly improves the dependability of the infrastructure chosen for green hydrogen production.

In this study eight critical parameters are included for mapping potential sites for green hydrogen production: distance to sea, elevation, land use/land cover, geology, slope, wind speed, distance to roads, and air temperature. The analytic hierarchy technique was used to determine appropriate weightings for parameters, resulting in the model including four distinct areas (least suitable, marginally suitable, moderately suitable, and most suitable) and suggesting the northern part as the most optimal location for green hydrogen production.

This study serves as a preliminary assessment and first step in the site selection process. Therefore, it is recommended that this analysis must be validated and refined through complementary studies like structural and engineering studies to know the nature of the lithological units and the assessment of bedrock and their extension in the regions and economic concerns to provide a more extensive appropriateness assessment. Furthermore, long-term data analysis and on-site inspections are vital for making educated decisions about the growth of green hydrogen production projects.

## Data Availability

The used datasets are publicly available through the following websites: 1. Land use/ land cover data at Esri Sentinel-2 Land Cover Explorer. 2. Air temperature data on WorldClim website. 3. Wind speed data at NASA website. 4. Geological data at USGS website, Elevation data available at many sources, such as USGS and Open Topography website. 5. Distance to roads at BBBike website. 6. Distance to sea was derived from the Marsa Matruh coastline shapefile downloaded from DIVA-GIS. Furthermore, we will be pleased to share any additional data or methodology upon request.
